# Park environment and moderate-to-vigorous physical activity in parks among adolescents in a high-density city: the moderating role of neighbourhood income

**DOI:** 10.1186/s12942-021-00289-7

**Published:** 2021-08-16

**Authors:** Ru Zhang, Chun-Qing Zhang, Poh Chin Lai, Wei Cheng, Benjamin Schüz, Mei-Po Kwan

**Affiliations:** 1 School of Physical Education and Sports Science, South China Normal University, Guangzhou, China; 2 Department of Psychology, Sun Yat-Sen University, No. 132 Waihuan East Road, Guangzhou Higher Education Mega Center, Guangzhou, China; 3 Department of Geography, Faculty of Social Sciences, The University of Hong Kong, Hong Kong, China; 4 Institute for Public Health and Nursing Research, University of Bremen, Bremen, Germany; 5 Department of Geography and Resource Management, The Chinese University of Hong Kong, Hong Kong, China; 6 Institute of Space and Earth Information Science, The Chinese University of Hong Kong, Hong Kong, China; 7 Department of Human Geography and Spatial Planning, Utrecht University, Utrecht, The Netherlands

**Keywords:** Urban parks, Moderate-to-vigorous physical activity, Park-based physical activity, Youth, Neighbourhood socioeconomic status

## Abstract

**Background:**

Urban parks are critical environmental resources in which adolescents engage in physical activity (PA). Evidence on the associations between park environmental characteristics and park-based PA in adolescents is mixed, particularly for high-density cities. Evidence is also lacking concerning the moderating role of neighbourhood socioeconomic status on the park-PA relationships. The current study aimed to examine the associations between park environmental characteristics and moderate-to-vigorous physical activity (MVPA) in parks among adolescents in Hong Kong and the moderating effect of neighbourhood income on these associations.

**Methods:**

A cross-sectional study involving direct observations of adolescents was conducted in 32 randomly selected urban parks in Hong Kong. Park environmental characteristics were measured using the Community Park Audit Tool. Park-based MVPA among adolescents was measured using the System for Observation Play and Recreation in Communities. Neighbourhood income was extracted from the 2011 Hong Kong Population Census data on median household income.

**Results:**

There was a significant positive association between the quality of amenities and park-based MVPA (metabolic equivalents per observation) in adolescents. However, the associations between the diversity of active facilities, greenness and adolescents’ park-based MVPA were not significant. Neighbourhood income moderated the association between adolescents’ park-based MVPA and park safety, where the relationship between park safety and park-based MVPA was significantly positive in low-income neighbourhoods but not significant in high-income neighbourhoods. An income-by-environment interaction was also observed concerning park aesthetics, with a negative relationship between park aesthetics and park-based MVPA in high-income neighbourhoods but not in low-income neighbourhoods.

**Conclusion:**

Our findings provide evidence regarding how park environment and neighbourhood income impact adolescents’ park-based MVPA in Hong Kong. These findings can inform urban planning and policymakers who seek to improve urban park development in high-density cities.

**Supplementary Information:**

The online version contains supplementary material available at 10.1186/s12942-021-00289-7.

As recommended by the World Health Organization, adolescents should engage in an average of 60 min of moderate-to-vigorous physical activity (MVPA) per day [1]. Health benefits of physical activity (PA) for adolescents are well established and include improved cardiorespiratory and musculoskeletal health and positive effects on cognitive development, mental health, and healthy weight [1, 2]. More importantly, the health benefits achieved during adolescence can carry forward into adulthood [3]. Nevertheless, most adolescents currently do not engage in sufficient PA to reap these benefits [4]. The global prevalence of insufficient PA is around 80% for adolescents aged 11–17 years [4]. In China, a national survey of 71,904 Chinese adolescents found that 70% of the adolescents were insufficiently physically active [5]. These findings point to an urgent need to develop evidence-based policies to promote MVPA in adolescents. One promising strategy is to improve urban environments such as urban parks to facilitate recreational PA [6].

Urban parks are critical environmental resources in which adolescents engage in PA [7]. With free and accessible PA facilities, urban parks are commonly available in neighbourhoods where adolescents live. Evidence for a positive relationship between park availability and leisure-time PA and total PA has been widely reported in adolescents [8]. For example, one study found that adolescents living in neighbourhoods with a higher number of urban parks were more likely to use parks for PA and perform more high-intensity PA than those with fewer parks in their neighbourhoods [9]. Access to urban parks also has a positive influence on increasing park use and PA in adolescents [7]. The impact of exposure to urban parks on increasing MVPA in adolescents was highlighted in studies demonstrating the positive association between park visits and adolescents’ total MVPA [10, 11].

Although previous studies showed that providing urban parks in neighbourhoods potentially increases park-based PA and promotes regular PA [7, 12], current evidence is mixed concerning the associations between park environmental characteristics and park-based PA in adolescents [7]. Some studies indicated that the number of park features (e.g., supporting amenities and PA facilities), greenness, park safety, and aesthetics were positively associated with self-reported and objectively-measured park-based PA among adolescents [13–15]. For example, one study found that park maintenance was one of the most crucial park characteristics for park-based PA, followed by the availability of park features such as playgrounds and sports fields [16]. In contrast, other studies suggested that the number of PA facilities, park aesthetics, and park safety were not associated with park-based PA among adolescents [17, 18]. Apart from the mixed evidence for park environmental characteristics, most studies relied primarily on self-reported scales to investigate PA in parks, which may introduce recall biases [7]. Unlike self-reported measures, momentary time sampling used in systematic observations is an effective strategy to record park users’ PA and their sociodemographic characteristics such as gender and age [19]. More empirical evidence is needed to show how adolescents use urban parks for PA and what park environmental characteristics may influence their active park use using momentary time sampling methods.

In light of the increasing evidence suggesting socioeconomic disparities in park environmental characteristics [20] and park-based PA in adolescents [9, 21, 22], an important question is whether neighbourhood socioeconomic status (SES) would modify the strength of the associations between park-based PA and park environmental characteristics. To the best of our knowledge, only a few studies have examined the moderating effects of neighbourhood SES on the park-PA relationship, and these studies did not reach consistent conclusions. For example, one study found a significant association between park availability and individuals meeting PA guidelines in low-income neighbourhoods but not in high-income neighbourhoods [23]. Other studies found that people living in low-SES neighbourhoods had superior access to public open spaces but were less likely to use these settings for PA than those living in high-SES areas [21, 24]. In contrast, other studies failed to identify a significant moderating effect of neighbourhood SES on the associations between park-based PA and park environmental characteristics [12, 25]. Regarding how neighbourhood SES influences the relationship between park environment and park-based PA, these inconsistencies suggest the need for further research. To inform future research addressing the inequality of PA among adolescents living in high and low SES areas, the moderating role of neighbourhood SES on the park-PA relationship needs to be further examined.

High-density cities are often characterised by mixed land use, high street connectivity, and adequate access to services and facilities such as urban parks [26, 27]. High-density urban areas are also associated with limited open spaces, environmental noise, and an increased desire for interaction with nature [28, 29]. To satisfy residents’ needs for leisure and recreation in their living areas, maintaining urban park environment has been proven to be an effective strategy for high-density cities [27]. Self-reported measures are commonly used in high-density cities in Asia such as Taipei, Hong Kong, and Beijing to examine park environmental characteristics [30]. Considering that there is a weak agreement between objectively-measured and perceived park environment [31, 32], these studies may provide biased estimates of the park-PA relationships in high-density cities [33]. Therefore, objectively-measured park environmental characteristics influencing park use for PA have not been thoroughly examined in high-density cities and warrant further research [34]. A solid understanding of the park-PA relationships in high-density cities is essential to provide empirical evidence to inform urban planners and policymakers who seek to promote PA in such geographic areas [35].

Therefore, the current study aimed to examine the associations between park environmental characteristics and park-based MVPA among adolescents in Hong Kong as an example of a high-density city and the moderating effect of neighbourhood income on these associations. We hypothesised that park environmental characteristics would be positively associated with park-based MVPA among adolescents [7]. These park environmental characteristics include the diversity of active facilities (e.g., tennis courts), quality of amenities (e.g., picnic tables), park safety, park aesthetics, and greenness. We also hypothesised that neighbourhood income would moderate the associations between park-based MVPA and park environmental characteristics [23].

## Methods

### Study design and setting

A cross-sectional study involving direct observations of adolescents was conducted in 32 randomly selected urban parks in Hong Kong, which is a high-density city with a population of 7.51 million in 2019 and a land area of approximately 1081 km^2^ [36, 37]. There were 209 Tertiary Planning Units (TPUs) in Hong Kong, representing the smallest census-based units associated with the Hong Kong 2011 Population Census data [38]. The 2011 census data were used because these data included the latest census results during the study period in 2018. We located 147 urban parks among the 209 TPUs.

A two-stage stratified sampling strategy based on TPU-level walkability and SES was used to randomly select a representative sample of urban parks in Hong Kong [39]. The TPU-level walkability was calculated as the sum of the standardised values (z-scores) of net residential density, land use mix, and intersection density [39]. The three components of the walkability index were measured using ESRI ArcGIS 10 (Redlands, CA; ESRI Inc, 2010). The TPU-level SES was calculated as the sum of the standardised values (z-scores) of the proportion of (a) single-person households; (b) never-married status; (c) low educational attainment; (d) households with low income; (e) unemployment rate; (f) adults with non-professional jobs; and (g) subtenancy. Data on the seven socioeconomic characteristics were extracted from the Hong Kong 2011 Population Census data [38]. These seven socioeconomic characteristics were selected because they are important factors used in the Hong Kong Population Census to profile area-level socioeconomic circumstances [40]. The TPU-level walkability and SES were classified into low (up to the 5th tertile) and high levels (above the 5th tertile). According to Kreft and De Leeuw [41], 30 groups with at least 30 individuals in each group is the smallest acceptable sample size for the estimation of the regression coefficients. Therefore, a total of 32 urban parks were randomly selected from the four walkability-by-SES categories (high SES/high walkability, high SES/low walkability, low SES/high walkability, and low SES/low walkability), with eight parks in each category. Only accessible parks with different park sizes and facilities were selected [42, 43].

### Study procedure

Before data collection, we prepared observation maps for the 32 urban parks and conducted training for observers. All potential areas in an urban park that could be used for PA were selected as activity areas. The activity areas were coded and marked in the observation map. The training involved a 2-day workshop and a 2-day field-based observation designed to teach the observers to collect data on park environmental characteristics and park-based PA using observation tools. We also trained the observers to distinguish adolescents from children and adults by observable physical and biological features [14]. A high level of agreement (intraclass correlation = 0.75 for age, gender, and activity levels) between the observers was required after the training [19]. Data on park environmental characteristics and park-based PA were collected by the trained observers between August 2018 and March 2019. Park environmental characteristics were measured using the Community Park Audit Tool [44]. Park-based PA was measured using the System for Observation Play and Recreation in Communities (SOPARC) at four time periods (7:30 am, 11:30 am, 3:30 pm, and 6:30 pm) on two weekdays and two weekend days [18, 40, 42].

### Measures

*Park environmental characteristics* The Community Park Audit Tool (CPAT) was used to measure four park environmental characteristics at the park level, including the diversity of active facilities, the quality of park amenities, park safety, and park aesthetics [44]. Specifically, the diversity of active facilities was measured as the sum of the presence (1 = presence or 0 = not presence) of activity areas used for active recreation and sports games (e.g., playground, fitness corners, and tennis courts). The quality of supporting amenities was measured as the sum of the usability (1 = all or most are usable or 0 = only about half or few are usable) and good condition (1 = all or most are in good condition or 0 = only about half or few are in good condition) of supporting amenities (e.g., restrooms, drinking fountains, and benches). Park safety was measured as the sum of the presence (1 = presence or 0 = not presence) of lights, park monitors, emergency devices, park visibility, and other safety concern (such as the presence of dangerous spots, threatening persons, and graffiti). Park aesthetics were measured as the sum of the presence (1 = presence or 0 = not presence) of landscaping, artistic features, historical/educational features, wooded areas, trees, water features, and meadows. Greenness areas were assessed using Normalized Difference Vegetation Index. Greenness percentage was calculated as greenness areas divided by park size [45]. Coding and scoring of these characteristics are shown in Additional file 1: Table S1.

Park-based MVPA among adolescents was measured using SOPARC, which is an observation tool for assessing PA in recreational places such as urban parks [19]. In each park, systematic observations were conducted by scanning from left to right. Separate observation scans were conducted for girls and boys and different activity types. We recorded the number of adolescents being sedentary and active during an observation scan. Inter-rater agreement for gender was 99%, and intraclass correlations for the number of sedentary and active park users were 0.95 and 0.98, respectively. During observation in parks, we identified activity types (e.g., walking and jogging) and coded them as light (e.g., walking in normal speed), moderate (e.g., fast walking), or vigorous (e.g., jogging) PA based on their MET values [46], where intensity levels of PA are coded as light (1.6–2.9 METs, e.g., stretching exercise), moderate (3–5.9 METs, e.g., fast walking), or vigorous (≥ 6 METs, e.g., jogging). This coding method was previously used [32, 47] and reached an acceptable inter-rater agreement (93%) in the current study. Park-based MVPA (METs per observation) was then calculated as the number of adolescents engaging in moderate PA × 3 METs + the number of adolescents engaging in vigorous PA × 6 METs [48, 49].

*Moderator*. Neighbourhood income was measured as a moderator of the association between park environment and park-based MVPA among adolescents. Neighbourhoods were defined as the 400 m street-network buffers surrounding an urban park. We used the 400-m street-network buffers because this distance is considered walkable for neighbourhoods in high-density cities such as Hong Kong [39]. Neighbourhood income (HK$) was extracted from the 2011 Hong Kong Population Census data on median household income [40].

*Covariates* Gender, time periods, day types, temperature, the formality of PA, supervision in PA, park size, neighbourhood walkability, and neighbourhood quality were measured as covariates because these variables can potentially influence the park-PA relationship [7, 9, 13]. Following the manual of SOPARC, we recorded time periods (7:30 am, 11:30 am, 3:30 pm, and 6:30 pm), day types (weekdays and weekend days), and temperature in each observation period. We also recorded gender, the formality of PA (individual, informal group activities, or formally organised events), and supervision in PA (no supervision, supervised by teachers/coaches, or supervised by parents/guardians/caregivers) during an observation scan. Park size was objectively measured using ESRI ArcGIS 10 (Redlands, CA; ESRI Inc, 2010). GIS data were also used to objectively measure the neighbourhood walkability index within the 400-m street-network buffers surrounding an urban park. The neighbourhood walkability index was the sum of z-scores of population density, land use mix, and intersection density [39]. Neighbourhood quality was measured as the sum of the presence (1 = presence or 0 = not presence) of park entry points, public transit stop, parking areas, bike routes, and traffic signals, safety, and aesthetics surrounding an urban park [44].

### Statistical analyses

Descriptive statistics including mean, standard deviation (*SD*), and percentages were evaluated first. Considering the hierarchical nature of the data (individual-level outcomes nested within urban parks), linear mixed-effects models were used to examine the associations between park environment and adolescents’ park-based MVPA and the moderating effect of neighbourhood income on the associations. Analyses were conducted in four steps. First, we estimated a null model (intercept-only model) to examine the effects of the cluster variable (i.e., urban park) on the dependent variable (i.e., MVPA in parks among adolescents). The intraclass correlation coefficient (ICC) was calculated as the park variance component divided by the total variance. Second, we estimated the associations between park environment and MVPA in parks among adolescents after adjustment for the covariates (Model 1 in Table 2). Third, we added two-way interaction terms to Model 1 to examine the moderating effects of neighbourhood income on the association between park environment and MVPA in parks among adolescents (Model 2 in Table 2). Finally, simple slope analyses were conducted for significant interaction terms by estimating associations at Mean ± 1SD for neighbourhood income. Likelihood ratio tests were used to compare model fit between the null model and Models 1 and 2. For all models, parameter estimates were generated using maximum likelihood estimation, and statistical significance was set at *p* < 0.05. All analyses were performed using STATA/SE 16 (StataCorp, College Station, TX, USA).

## Results

### Descriptive results

Table 1 presents descriptive characteristics of the study participants, park environment, and surrounding neighbourhoods. Overall, 3368 adolescents were observed in the 32 urban parks in Hong Kong, of which 583 were sedentary, and 2785 were active during observation. Of the active adolescents, 2397 were observed using urban parks for MVPA and were included for further analyses. The participants were predominantly boys (83.5%), and most adolescents used urban parks in the evening (49.5%) and weekends (63.1%). The majority were observed using urban parks for informal group activities (92.2%) and were not under the supervision of teachers or guardians (85.9%). On average, the participants engaged in MVPA equivalent to 15.86 METs (*SD* = 19.48) per observation. The mean temperature during the observation period was 24.56 °C (*SD* = 4.76). The average size of the 32 urban parks was 8.58 hectares (*SD* = 7.19), and each urban park contained an average of 7.74 types (*SD* = 2.57) of active facilities. Neighbourhoods within the 400-m buffers surrounding an urban park were characterised by an average of HK$25,000 (*SD* = 8400) median household income.

**Table 1:** Descriptive characteristics of covariates, park environment, neighbourhood income, and park-based MVPA among adolescents (*n* = 2397)

Variables	*n* (%) or *M* (*SD*)
*Covariates*	
Gender	
Girls	396 (16.5%)
Boys	2001 (83.5%)
Time periods	
Morning	119 (5.0%)
Noon	348 (14.5%)
Afternoon	744 (31.0%)
Evening	1186 (49.5%)
Day types	
Weekdays	885 (36.9%)
Weekend days	1512 (63.1%)
Formality of physical activity	
Individual	169 (7.1%)
Informal group activities	2212 (92.2%)
Formally organized events	16 (0.7%)
Supervision in physical activity	
No supervision	2058 (85.9%)
Teachers/coaches	290 (12.1%)
Parents/guardians/caregivers	49 (12.0%)
Temperature (℃)	24.56 (4.76)
Neighbourhood quality ^a^	8.07 (1.08)
Walkability	− 0.23 (1.29)
Park size (ha)	8.58 (7.19)
*Park environmental characteristics*^b^	
Diversity of active facilities	7.74 (2.57)
Quality of supporting amenities	7.15 (1.34)
Park safety	3.85 (0.84)
Park aesthetics	2.96 (1.61)
Greenness (%)	57.86 (21.33)
Neighbourhood income (HK$ 10 K)	2.50 (0.84)
Park-based MVPA (METs per observation)	15.86 (19.48)

*HK$* Hong Kong dollar, *ha* hectare, *MET*  metabolic equivalent, *MVPA*  moderate-to-vigorous physical activity, *M*  mean, *SD*  standard deviation, ℃  Degree Celsius

^a^The scores of neighbourhood quality ranged from 0 to 16

^b^The scores of the diversity of active facilities ranged from 0 to 16, the scores of the quality of supporting amenities ranged from 0 to 20, the scores of park safety ranged from 0 to 7, the scores of park aesthetics ranged from 0 to 7

### Park environment and park-based MVPA

Estimates of the null model indicated that 12.1% of the variance in park-based MVPA among adolescents was due to unmeasured park-level factors (ICC = 0.121, χ^2^ (1) = 70.51, *p* < 0.001), suggesting that park-based MVPA among adolescents varied across urban parks. Park environmental characteristics, neighbourhood income within the 400-m buffers from urban parks, and covariates were then entered in the null model (Model 1 in Table 2). Model 1 showed a better model fit than the null model (χ^2^ (24) = 326.01, *p* < 0.001). After adjusting for covariates and neighbourhood income, adolescents’ park-based MVPA had a positive association with the quality of amenities (*β* = 1.90, *p* = 0.025) and park safety (*β* = 4.15, *p* < 0.001), while adolescents’ park-based MVPA was negatively associated with park aesthetics (*β* = − 1.94, *p* = 0.017). There were no significant associations between adolescents’ park-based MVPA and the diversity of active facilities and greenness in parks.

**Table 2:** Estimates for models of the associations between park environment, neighbourhood income, and park-based MVPA among adolescents (*n* = 2397)

Parameter	Model 1				Model 2			
	Estimate	SE	95% CI	*p*	Estimate	SE	95% CI	*p*
*Fixed effects*								
Covariates								
Gender (ref = Boy)	− 6.98	1.58	− 10.08 to − 3.89	< .001	− 6.88	1.57	− 9.96 to − 3.81	< .001
Time periods (ref = Morning)								
Noon	− 1.22	3.12	− 7.33 to 4.88	.695	− 2.18	3.11	− 8.27 to 3.91	.483
Afternoon	1.12	2.99	− 4.74 to 6.97	.709	− 0.42	2.99	− 6.29 to 5.44	.887
Evening	6.34	2.91	0.64 to 12.03	.029	5.06	2.90	− 0.62 to 10.74	.001
Week types (ref = Weekday)	3.16	1.44	0.32 to 5.99	.029	2.85	1.43	0.05 to 5.65	.046
Formality of PA (ref = Individual)								
Informal group activities	9.46	1.78	5.97 to 12.96	< .001	9.68	1.77	6.21 to 13.15	< .001
Formally organized events	42.72	12.32	18.57 to 66.86	.001	41.56	12.15	17.75 to 65.38	.001
Supervision in PA (ref = No supervision)								
Teachers/coaches	7.28	2.57	2.26 to 12.31	.005	7.32	2.56	2.30 to 12.35	.004
Parents/guardians/caregivers	− 12.18	3.32	− 18.68 to − 5.68	< .001	− 10.70	3.27	− 17.11 to − 4.29	.001
Temperature	− 0.30	0.24	− 0.78 to 0.17	.204	0.18	0.30	− 0.41 to 0.76	.552
Neighbourhood quality	0.96	1.02	− 1.04 to 2.96	.349	3.06	1.94	− 0.74 to 6.87	.115
Walkability	0.67	0.86	− 1.01 to 2.35	.437	2.14	1.31	− 0.44 to 4.71	.104
Park size	0.24	0.21	− 0.17 to 0.66	.250	− 0.28	0.27	− 0.82 to 0.25	.299
Park environment								
Diversity of active facilities	− 0.92	0.62	− 2.12 to 0.29	.135	− 2.29	1.65	− 5.52 to 0.94	.165
Quality of supporting amenities	1.90	0.85	0.23 to 3.56	.025	3.15	3.58	− 3.87 to 10.16	.379
Park safety	4.15	1.19	1.82 to 6.48	< .001	17.45	5.94	5.80 to 29.10	.003
Park aesthetics	− 1.94	0.81	− 3.52 to − 0.35	.017	4.53	3.71	− 2.75 to 11.81	.223
Greenness	− 0.72	0.05	− 0.16 to 0.02	.140	− 0.32	0.23	− 0.78 to 0.14	.179
Neighbourhood income	− 3.02	1.20	− 5.36 to − 0.67	.012	19.08	15.67	− 11.63 to 49.79	.223
Interaction terms								
Diversity of active facilities × Neighb. income					0.39	0.58	− 0.74 to 1.52	.497
Quality of supporting amenities × Neighb.income					0.15	1.48	− 2.76 to 3.05	.920
Park safety × Neighb. income					− 5.27	2.08	− 9.36 to − 1.19	.011
Park aesthetics × Neighb. income					− 3.24	1.50	− 6.18 to − 0.30	.031
Greenness × Neighb. income					0.10	0.08	− 0.07 to 0.26	.241
*Random variance*^*a*^								
Residual	289.83	16.21	259.74 to 323.41	–	281.04	15.31	252.58 to 312.70	–
Intercept (Urban parks)	1.74	4.67	0.01 to 329.68	–	1.08e−14	1.07e−13	4.31e−23 to 2.70e−06	–
*Goodness of fit*								
−2 Log Likelihood	5737.42				5,713.07			

*95% CI*  95% confidence interval, *MVPA*  moderate-to-vigorous physical activity, *Neighb.income*  neighbourhood income, *PA* physical activity, *SE*  standard error

^a^In the null model, −2 Log Likelihood = 6063.43. Variance _Park_ = 45.02, 95% CI = [21.79, 93.04]. Variance _Residual_ = 326.32, 95% CI = [293.40, 362.94]. The intraclass correlation coefficient (ICC) = 45.02/ (45.02 + 326.32) = 0.121

– Since zero was not within its 95% CI, the statistical significance was below than .05

### Moderating effect of neighbourhood income

Model 2 in Table 2 represents the moderating effect of neighbourhood income on the associations between park environmental characteristics and park-based MVPA among adolescents. After two-way interaction terms were added in Model 2, the model fit was significantly better than Model 1 (χ^2^ (5) = 24.35, *p* < 0.001). Adolescents’ park-based MVPA was significantly associated with park safety × neighbourhood income (*β* = − 5.27, *p* = 0.011) and park aesthetics × neighbourhood income (*β* = − 3.24, *p* = 0.031), while the associations between adolescents’ park-based MVPA and other interaction terms were not significant.

**Fig. 1:**
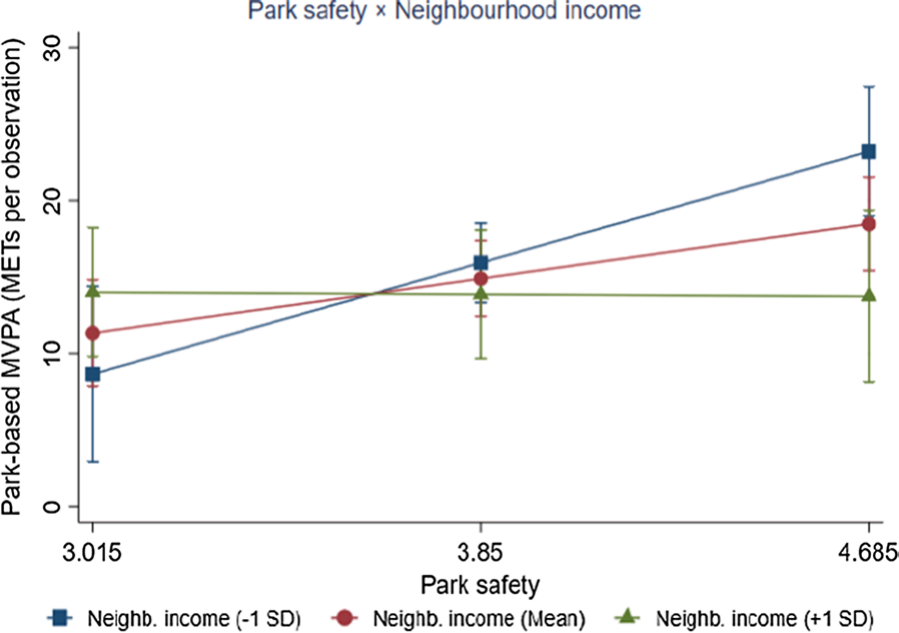
Moderating effect of neighbourhood income on the association between adolescents’ park-based moderate-to-vigorous (MVPA) and park safety

**Fig. 2:**
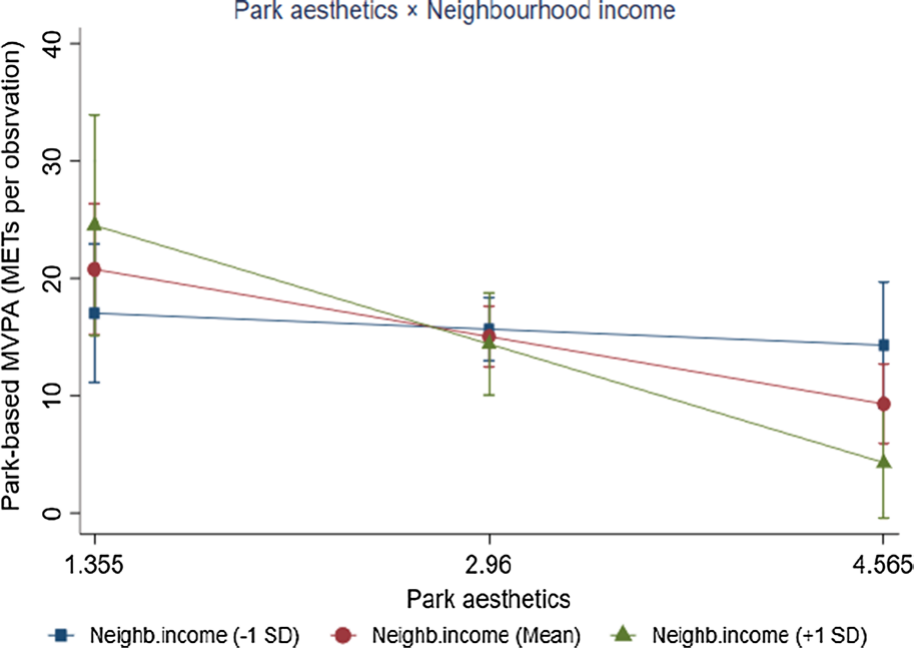
Moderating effect of neighbourhood income on the association between adolescents’ park-based moderate-to-vigorous (MVPA) and park aesthetics

We illustrated the associations of park-based MVPA with park safety and park aesthetics (Mean ± 1SD) for neighbourhood income (Figs. 1, 2). For neighbourhoods with below-average household income, there was a positive association between park safety and adolescents’ park-based MVPA (*β* = 8.71, *p* = 0.001), while the association was not significant for neighbourhoods with high-average household income (*β* = − 0.16, *p* = 0.921). A negative association between adolescents’ park-based MVPA and park aesthetics was only observed in higher-income neighbourhoods (*β* = − 6.30, *p* = 0.001) but not in neighbourhoods with below-average household income (*β* = − 0.85, *p* = 0.590).

## Discussion

Using Hong Kong as an example of a high-density city, the current study examined the associations between park environmental characteristics and adolescents’ park-based MVPA and the moderating effect of neighbourhood income on these associations. Significant associations were found between park-based MVPA and the quality of supporting amenities, park safety, and park aesthetics, while the association was not significant for the diversity of active facilities or greenness. Neighbourhood income moderated the associations of adolescents’ park-based MVPA with park safety and park aesthetics. These findings partially support our hypotheses and provide evidence for the impact of park environment and neighbourhood income on adolescents’ park-based MVPA, which can be used to inform urban planners and policymakers to improve urban park development in high-density cities.

The quality of park amenities is positively related to adolescents’ park-based MVPA in Hong Kong, suggesting that adolescents are likely to engage in MVPA in parks with well-maintained amenities. This might be because Hong Kong is a high-density city with limited recreational spaces, and therefore urban parks remain one of the major public spaces where adolescents can engage in PA in their leisure time [50]. This finding also corroborates the supportive role of the usability and condition of park amenities in prolonging adolescents’ park-based MVPA. Indeed, adolescents may need park amenities such as drinking fountains, benches, and picnic tables to support them in using parks for PA for more extended periods [17]. Despite the evidence on the quality of park amenities supporting adolescents’ park-based MVPA, future research needs to examine the relationship between the quality of park amenities and MVPA in parks via natural experiments (e.g., longitudinal pre/post quasi-experiments).

The current study did not identify a significant association between park-based MVPA and the diversity of active facilities in adolescents. However, previous studies have shown that both active facilities and greenness are important park features that influence park visits and PA in parks [7]. This finding is in line with a previous study in which the availability and quantity of active facilities were not associated with park-based PA among children and adolescents [17]. A possible explanation is that compared with unrenovated park features, renovating and increasing active facilities in parks might be more relevant to young people who use parks for PA [13, 51]. For example, one study found that renovated facilities had stronger associations with PA among adolescents than unrenovated schoolyards [52]. Therefore, renovating park facilities regularly would appear to be an essential strategy for increasing adolescents’ park-based MVPA. We also found that greenness was not associated with park-based MVPA. This may be explained by the small sample size of parks and the lack of variety in greenness across the sample [17]. Some policies have been designed to rehabilitate greenness in Hong Kong. For example, the "Hong Kong 2030 Plus" strategy highlighted the need for increasing vegetation cover such as grasslands and woodlands within a 400-m neighbourhoods [53]. Such policies may improve equity in greenness across parks in Hong Kong [27].

Neighbourhood income moderated the association between park safety and park-based MVPA among adolescents. We found a positive association between park safety and adolescents’ park-based MVPA in low-income neighbourhoods, however, the association was not significant in high-income areas. It can be assumed that urban parks in high-income neighbourhoods may present sufficient safety equipment (e.g., lights and park monitors) and present little safety concern (e.g., presence of dangerous spots), suggesting a lack of variety in park safety across the urban parks in high-income neighbourhoods [20, 54]. In low-income neighbourhoods, adolescents’ park-based MVPA was positively associated with park safety, suggesting that safe urban parks can attract adolescents to use parks for PA in low-income neighbourhoods in Hong Kong. Urban parks in low-income neighbourhoods are more likely to have personal safety problems such as threatening persons than those in high-income neighbourhoods [55]. Overall, crime-related safety remains an essential issue that impacts park use in adolescents living in low-income neighbourhoods, and this result needs to be addressed in future strategies promoting safety [56].

Neighbourhood income moderated the association between park aesthetics and adolescents’ park-based MVPA. The aesthetics-MVPA relationship was negative in high-income neighbourhoods but not significant in low-income neighbourhoods. Although adolescents with a higher perception of neighbourhood aesthetics are more likely to engage in PA, mixed evidence has been found to support the association between park aesthetics and park-based PA [7, 57]. A possible explanation for our findings is that adolescents living in high-income neighbourhoods may consider an urban park with high levels of aesthetics (e.g., presence of artistic features, historical/educational features, water features, and wooded areas) as a landscape park instead of a setting for PA [57]. It is assumed that adolescents use these parks for recreational activities, rather than for MVPA. In addition, high-income neighbourhoods might provide a wide selection of PA settings for adolescents such as PA clubs, which are more attractive than urban parks [6]. While for those living in low-income neighbourhoods, urban parks are their major places for PA, regardless of park aesthetics. Therefore, our findings imply that park aesthetics are of limited relevance for adolescents’ park-based MVPA.

### Limitations and future directions

Limitations of the current study should be acknowledged. First, the current study did not include park facilities that were not free for public access or use when measuring the diversity of active facilities. Future research should examine how pay-for-use facilities impact adolescents’ park-based MVPA [14]. Second, the current study used CPAT to measure safety and aesthetics in urban parks in Hong Kong. Although CPAT has been widely used to assess the park environment and reached moderate-to-high reliability [44], some of its items concerning park safety (e.g., presence of graffiti) and park aesthetics (e.g., presence of animal waste) were not suitable for Hong Kong, where graffiti and animal waste were seldom observed. Future studies may develop new measures that are suitable for the geographical and cultural contexts of Hong Kong [31]. For example, emergency devices and pavilions are suitable items for assessing safety and aesthetics in urban parks in Hong Kong [47]. Like other park observation audit instruments, CPAT requires observers to observe and evaluate park environment. Assessment of park environment may be influenced by the observers’ perception and judgments, especially when measuring park feature quality (whether park features such as picnic tables are usable and well maintained). Accurately assessing the actual and total park environment remains elusive.

Third, the current study measured MVPA in parks using momentary time sampling of systematic observation but did not consider adolescents' daily mobility. To better understand how greenspace exposure influences park-based PA in space and time, future research needs to use accessibility measures that consider adolescents’ daily mobility and accelerometers to track their greenspace exposure and park-based PA [58]. Finally, most park users observed in the current study were boys (83.5%). The current study did not test how gender influences the association between park environment and adolescents’ park-based MVPA. Previous studies revealed gender differences in adolescents’ park-based MVPA [13, 18]. Therefore, future research should examine the moderating effect of gender on the park-PA relationship better to understand gender influences.

## Conclusions

The current study found that adolescents were likely to engage in park-based MVPA with well-maintained amenities. Compared with adolescents living in high-income neighbourhoods, park safety significantly impacted those living in low-income neighbourhoods. The diversity of active facilities, greenness, or park aesthetics appeared to be of limited relevance for adolescents’ park-based MVPA. These findings provide evidence on the impact of park environment and neighbourhood SES on adolescents’ park-based MVPA and contribute to urban planning and policies to improve sustainable development in park environment in high-density cities. To promote adolescents’ park-based MVPA, supporting amenities such as drinking fountains, benches, and picnic tables should be well-maintained. Safety issues in parks should also be addressed in future safety strategies, especially for deprived neighbourhoods.

## Supplementary Information

**Additional file 1: Table S1.** Measures, data sources, scoring, and reliability of park environmental characteristics, moderator, park-based MVPA, and covariates.

## Data Availability

The datasets used and/or analysed during the current study are available from the corresponding author on reasonable request.

## Abbreviations

CPAT: The Community Park Audit Tool
MET: Metabolic equivalent
MVPA: Moderate-to-vigorous physical activity
PA: Physical activity
SES: Socioeconomic status
SOPARC: The System for Observation Play and Recreation in Communities
