# Supplementary material for: Park environment and moderate-to-vigorous physical activity in parks among adolescents in a high-density city: the moderating role of neighbourhood income
Source: Int J Health Geogr. 2021 Aug 16;20:35. doi: 10.1186/s12942-021-00289-7 (PMC8365917; doi:10.1186/s12942-021-00289-7)
Supplement: Supplementary file 1 — Additional file 1: Table S1. Measures, data sources, scoring, and reliability of park environmental characteristics, moderator, park-based MVPA, and covariates. [file 12942_2021_289_MOESM1_ESM.docx]

| Table S1.  *Measures, data sources, scoring, and reliability of park environmental characteristics, moderator, park-based MVPA, and covariates* | | | | | |
| --- | --- | --- | --- | --- | --- |
| Variables | | | Measures/  Data sources | Scoring | Reliability (inter-rater agreement or intraclass correlation) |
| **Park environment** | | |  |  |  |
|  | Diversity of active facilities | | CPAT | The sum of the presence (1 = presence or 0 = not presence) of playgrounds, soccer pitches, swimming pools, basketball courts, tennis courts, volleyball courts, walking/jogging trails, fitness corners, skate parks, green spaces, gateball courts, cycling trails, badminton courts, table tennis courts, indoor sports centers, pebble trails | 75% |
|  | Quality of supporting amenities | | CPAT | The sum of the usability (1 = all or most are usable or 0 = only about half or few are usable) and good condition (1 = all or most are in good condition and 0 = only about half or few are in good condition) of restrooms, drinking fountains, benches, picnic tables, and trash cans. | 80% |
|  | Park aesthetics | | CPAT | The sum of the presence (1 = presence or 0 = not presence) of landscaping, artistic features, historical/educational features, wooded areas, trees, water features, and meadows. | 95% |
|  | Park safety | | CPAT | The sum of the presence (1 = presence or 0 = not presence) of lights, park monitors, emergency devices, park visibility, and other safety concern (such as the presence of dangerous spots, threatening persons, and graffiti). | 85% |
|  | Greenness | | NDVI | Greenness areas were measured using Normalized Difference Vegetation Index, greenness percentage was calculated as greenness areas divided by park size | ‒ |
| **Moderator** | | |  |  |  |
|  | | Neighbourhood  income | Census data | Neighbourhood income (HK$) was extracted from the 2011 Hong Kong Population Census data on median household income. Neighbourhoods were defined as the 400-m street-network buffers surrounding an urban park | ‒ |
| **Park-based MVPA** | | | SOPARC | Park-based MVPA (METs per observation) was calculated as the number of adolescents engaging in moderate PA × 3 METs + the number of adolescents engaging in vigorous PA × 6 METs | Inter-rater agreement for intensity levels: 93%  Intraclass correlation for number of active park users: r = 0.95 |
| **Covariates** | | |  |  |  |
|  | Gender | | SOPARC | Boy or girl | 99% |
|  | Time periods | | SOPARC | 7:30 am, 11:30 am, 3:30 pm, or 6:30 pm | ‒ |
|  | Day types | | SOPARC | Weekdays or weekend days | ‒ |
|  | Formality of PA | | SOPARC | Individual, informal group activities, or formally organised events | 99% |
|  | Supervision in PA | | SOPARC | No supervision, supervised by teachers/coaches, or supervised by parents/ guardians/ caregivers | 99% |
|  | Park size | | GIS | ‒ | ‒ |
|  | Temperature | | ‒ | ‒ | ‒ |
|  | Walkability | | GIS | The sum of the standardized values (z-scores) of net residential density, land use mix, and intersection density. | ‒ |
|  | Neighbourhood quality | | CPAT | The sum of the presence (1 = presence or 0 = not presence) of park entry points, public transit stop, parking areas, bike routes, traffic signals, safety, and aesthetics surrounding an urban park | ‒ |

*Note*. *CPAT*: Community Park Audit Tool. *GIS*: Geographic Information Systems databases. HK$: Hong Kong dollar. *MVPA*: Moderate-to-vigorous physical activity. NDVI: Normalized Difference Vegetation Index; *PA*: Physical activity *SOPARC*: System for Observation Play and Recreation in Community. *TPU*: Tertiary Planning Unit. ‒ : Not relevant
